# Successful live birth in a Chinese woman with P450 oxidoreductase deficiency through frozen-thawed embryo transfer: a case report with review of the literature

**DOI:** 10.1186/s13048-021-00778-0

**Published:** 2021-02-01

**Authors:** Ping Pan, Lingyan Zheng, Xiaoli Chen, Jia Huang, Dongzi Yang, Yu Li

**Affiliations:** Reproductive Medicine Centre, Department of Gynecology & Obstetrics, Sun Yat-sen Memorial Hospital, Sun Yat-sen University, 107 Yanjiang West Road, Guangzhou, 510120 China

**Keywords:** Congenital adrenal hyperplasia, P450 oxidoreductase deficiency, In vitro fertilization, Live birth

## Abstract

**Background:**

Congenital adrenal hyperplasia (CAH) caused by P450 oxidoreductase deficiency (PORD) in 46, XX patients is characterized by genital ambiguity, primary amenorrhea, absent or incomplete sexual maturation, infertility, skeletal malformations and so on. But few pregnancies have been reported from these female patients with PORD.

**Case description:**

A 29-year-old Chinese woman with PORD due to the compound heterozygous mutation (c.1370G > A/c.1196_1204del) in the P450 oxidoreductase (*POR*) gene had suffered from primary amenorrhea and infertility. She had one cancelled cycle of ovulation induction due to low serum estradiol(E_2_), high progesterone(P) levels and thin endometrium, then in vitro fertilization (IVF) was recommended. At the first IVF cycle, 4 oocytes were retrieved and 4 viable embryos were cryopreserved due to thin endometrium associated with low E_2_ and prematurely elevated P after ovarian stimulation, even though oral dexamethasone were used to control adrenal P overproduction at the same time. When basal P fell to < 1.5 ng/ml after the therapy of oral dexamethasone, artificial endometrial preparation and frozen embryo transfer were performed, resulting in a twin pregnancy. She delivered a healthy boy and a healthy girl by caesarean section at 37 weeks and 2 days of gestation. After the literature search in PORD women, no spontaneous pregnancy has been reported and only two previous case reports of 3 successful pregnancies through IVF were summarized.

**Conclusions:**

It is the third report that successful pregnancy was achieved in a CAH woman caused by a compound heterozygous *POR* mutation, with primary amenorrhea and disorders of steroidogenesis. It seemed that disorders of steroidogenesis caused by PORD didn’t impair the developmental potential of oocytes. IVF and frozen embryo transfer after adequate hormonal control and endometrial preparation should be an effective infertility treatment for PORD women.

## Background

The enzyme P450 oxidoreductase (POR) is encoded by the *POR* gene on chromosome 7 [17]. POR transfers electrons from reduced nicotinamide adenine dinucleotide phosphate (NADPH) to all microsomal (type II) cytochrome P450 enzymes, including three steroidogenic enzymes: P450c17 (17α-hydroxylase/17,20 lyase), P450c21 (21-hydroxylase), and P450aro (aromatase) [18, 21].

P450 oxidoreductase deficiency (PORD) is a rare autosomal recessive variant of congenital adrenal hyperplasia (CAH) arising from homozygous or compound heterozygous *POR* gene mutations. In 2004, mutations in *POR* gene disrupting steroid biosynthesis were firstly reported [12, 15]. Up to now over 100 cases and more than 50 different *POR* mutations have been reported (7). Patients with PORD occur mostly in neonates and children, and have a range of skeletal malformations, glucocorticoid deficiency and disorders of sexual development (DSD) [7]. Although one pair of *POR* mutations can impair all microsomal cytochrome P450 enzymes, each enzyme is affected to a different extent (depending on the locations of the *POR* gene mutations), resulting in high clinical variability of PORD, such as it has been reported that young girls or women only had incomplete pubertal development, primary amenorrhea, oligomenorrhea or infertility with or without skeletal malformations [2, 23, 25]. The clinical course of PORD in adulthood and the long-term consequence for female fertility remain unknown. In theory, the female fertility should be severely impaired by the presence of DSD and disordered steroidogenesis due to reduced activities of three steroidogenic enzymes caused by PORD [3].

We report a live birth from a Chinese woman who presented with primary amenorrhea and infertility caused by a compound heterozygote *POR* mutation.

## Case

To report this case, appropriate written consent and assent had been obtained in accordance with the guidelines of the ethics committee of Sun Yat-sen Memorial Hospital, Sun Yat-sen University (SYSEC-KY-KS-2019-052).

### Clinical and biochemical presentation

The patient was born at term after a normal pregnancy and delivery. Her parents were nonconsanguineous and she had a healthy and fertile elder brother. At birth, she was healthy and had external female genitalia. At 16 years old, she presented with normal breast development, no pubic or axillary hair, normal blood pressure, but no menses, and she was evaluated for primary amenorrhea by a local gynecologist. Her karyotype was 46, XX and a pelvic ultrasound revealed the presence of 4 × 3 × 4 cm ovarian cyst in the left ovary and an infantile uterus (hormonal data are not available). However the etiology of her amenorrhea remained unknown at that time. After that, she had accepted hormone replacement therapy (HRT) to establish a regular menstrual cycle but her menses didn’t come when she stopped HRT. When she was 29 years old and had suffered from primary infertility for 3 years, she was referred to treat infertility and she had a cancelled cycle of ovulation induction in the local hospital. The follicle growth and sex hormone changes during the ovulation induction were as the following: human menopausal gonadotropin (hMG)(150 IU/d) were administered for 17 days from the cycle 3 of inducing menstruation after two-month oral contraception pills, and two follicles grew to 18 mm and 17 mm in size but serum E_2_ level remained very low (< 5 pg/ml) with P level increasing to 25.1 ng/ml and a thin endometrium (3 mm). Ovulation trigger was cancelled due to the thin endometrium and the abnormal levels of E_2_ and P.

Then she was referred to Reproductive Medicine Center of Sun Yat-Sen Memorial Hospital, Sun Yat-Sen University in 2014, willing to have a child. Physical examination revealed the following characteristics: a height of 158 cm and weight of 60 kg; Tanner scores of four for the breasts and two for axillary and pubic hair; female external genitalia; difficulty of bending the metacarpopha-langeal joints from childhood; no other skeletal malformations were founded. No other infertility factor was identified. The evaluations for adrenal, gonadal and pituitary hormones showed that serum levels of P and 17-hydroxyprogesterone (17-OHP) were obvious high, and dehydroepiandrosterone sulfate (DHEA-S), androstenedione, free testosterone were low, as well as the other tests were within the reference ranges. Table 1 summarized the clinical characteristics and hormonal profiles of the patient. A pelvic ultrasonography showed a hypoplastic uterus, thin endometrium and an ovarian cyst (2.9 × 3.0 × 2.8 cm) in the right ovary. Bilateral integration of the adrenal glands was enlarged, as determined by a computed tomography scan.

**Table 1:** Clinical Characteristics and Hormonal Profiles of the study patient

	Case
Age (years)	29
Menstruation	Primary amenorrhea
Height (cm)	158
Weight (kg)	60
BMI (kg/m^2^)	24.03
Blood pressure (mmHg)	118/79
Karyotype	46XX, 1qh+
Antral follicle count (n)	6
AMH (ng/ml)	2.53
FSH (IU/L)	14.80
LH (IU/L)	8.84
E_2_ (pg/ml)	21
Prolactin (IU/L)	20.9
Testosterone (nmol/L)	1.21
Progesterone (ng/ml)	> 40.1
17-OHP (ng/ml)	> 20
Free testosterone (pg/ml)	0.19
DHEA-S (ng/ml)	223.99
Androstenedione (ng/ml)	0.82
SHBG (nmol/L)	43.39
TSH (mIU/L)	2.61
ACTH (pg/ml) 8:00 a.m	140.00
ACTH (pg/ml) 4:00 p.m	21.00
Cortisol (nmol/L) 8:00 a.m	474.39
Cortisol (nmol/L) 4:00 p.m	271.82
Aldosterone (ng/L)	115.51
Serum potassium (mmol/L)	4.23
Serum sodium (mmol/L)	141.9
Renin concentration (ng/ml/h)	2.95

*Abbreviation*: *BMI* Body mass index, *AMH* Anti-Müllerian hormone, *FSH* Follicle-stimulating hormone, *LH* Luteinizing hormone, *E*_*2*_ Estradiol, *17-OHP* 17α-hydroxyprogesterone, *DHEA-S* Dehydroepiandrosterone sulfate, *SHBG* Sex hormone-binding globulin, *TSH* Thyroid stimulating hormone, *ACTH* Adrenocorticotropic hormone

### Genetic testing

The patient was suspected of having rare forms of CAH according to the clinical manifestations, imaging and laboratory tests. In order to confirm the diagnosis and find the genetic etiology, a panel of CAH candidate genes by targeted exome next-generation sequencing (NGS) were performed, including *CYP21A2*, *CYP19A1*, *CYP17A1, CYP11A1, HSD3B2, STAR, AR, EDNRA, NR5A1, PDE8B* and *POR* gene.

Genomic DNA were extracted from the peripheral blood leukocytes using the QIAamp DNA Blood Mini Kit (Qiagen, Hilden, Germany). The extracted DNA was segmented by DNA enzyme and purified by magnetic bead (Beckman Inc., USA), followed by PCR amplification. DNA library was captured and purified twice by a customized Panel probe (Illumina Inc., USA). The exon, intron-exon boundaries, the 5’and 3′ flanking regions of the panel genes was sequenced by NextSeq500 (Illumina Inc., USA).

Raw data was compared with reference sequence retrieved from the University of California at Santa Cruz Genome Browser (http://genome.ucsc.edu) (UCSC, hg19) by the BWA algorithm and annotated using the method reported by Zhang [29]. The HGVS (www.hgvs.org/mutnomen/) guidelines for describing sequence variations and numbering were used, with + 1 corresponding to the A of the ATG translation initiation codon of the GenBank cDNA sequence and the amino acid sequences. All variants were classified according to the American College of Medical Genetics and Genomics (ACMG) 2015 classification [24]: pathogenic, likely pathogenic, uncertain significance, likely benign and benign. Sanger sequenced was performed in suspected variations.

**Fig. 1:**
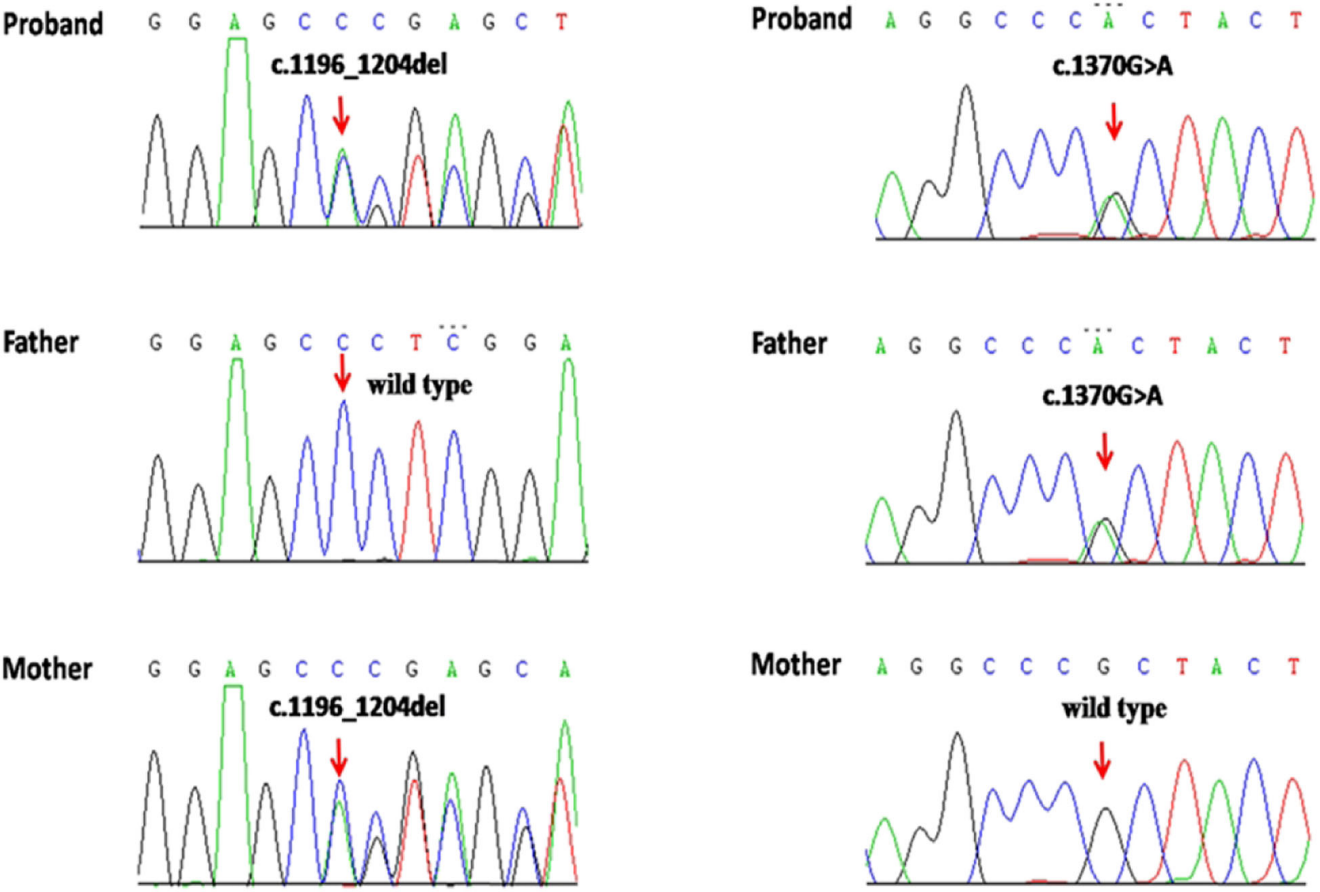
The Sequencing chromatogram of the mutations from the proband patient and her parents

The results showed that no mutation and copy number variation were found in *CYP21A2, CYP19A1*, *CYP17A1, CYP11B1, HSD3B2, AR, EDNRA, NR5A1, PDE8B* and *STAR*, but a compound heterozygous mutation was found in *POR* gene (NM_000941.2): c.1370G > A (p.Arg457His, rs28931608) and c.1196_1204del (p.Pro399_Glu401del) (Fig. 1). The sequencing results of her parents showed that her father was a heterozygous carrier for c.1370G > A and her mother was a heterozygous carrier for c.1196_1204del. The c.1370G > A had been found in some PORD patients (HGMD:CM040474), which are common in Japanese and Chinese patients [2, 7, 9, 12, 13]. The mutation of c.1370G > A in *POR* gene leads to a conversion of arginine at amino acid position 457 to histidine (R457H) which supports only 3% of 17-hydroxylase activity, no detectable 17,20 lyase activity [12, 15], and only 1% of aromatase activity [22]. The c.1196_1204del mutation in *POR* gene was firstly reported in two unrelated Turkish PORD patients (HGMD ID:CD117091) and cause a loss of three amino acid p.Pro399_Glu401del (P399_E401del) [11] . In comparison to wild-type POR, this P399_E401del mutation was found to decrease catalytic efficiency of 21-hydroxylase by 68%, 17α-hydroxylase and 17,20 lyase by 76 and 69%, and aromatase by 85% [5, 11]. The variants c.1370G > A and c.1196_1204del were classified by pathogenic and likely pathogenic respectively according to ACMG.

### Diagnosis and differential diagnosis

The elevation of serum basal morning 17OHP concentration is usually used to diagnosis the other types of CAH such as 21-hydroxylase or 11β-hydroxylase deficiency, while the major clinical presentation in these two types of CAH are atypical genitalia, precocious pubarche, hirsutism, oligomenorrhea/amenorrhea and without sex steroid deficiency and skeletal malformation [8]. Our case presented with no signs of virilization or clinical/biochemical hyperandrogenism, but impaired estradiol production, primary amenorrhea and minor skeletal malformation. Those are the clinical features of PORD [8]. Additional genetic testing also confirmed the PORD diagnosis.

### Fertility treatment

She was given HRT composed of estradiol and progesterone (Femoston, Abbott Biologicals B. V, Netherland), combined with oral dexamethasone (0.375 mg/d) for 2 months. After the therapy her basal serum P and 17-OHP levels fell to normal levels (0.35 ng/ml and 0.23 ng/ml respectively) with disappearance of the ovarian cyst. According to our several successful cases with atypical CAH caused by 17α-hydroxylase deficiency through frozen embryo transfer after IVF and the pregnant case with 17α-hydroxylase deficiency published in 2016 [4] who showed similar changes of sex hormones and inadequate endometrial development during ovarian stimulation, IVF management was recommended. Oral dexamethasone (0.375 mg/d) was maintained during all treatment phases.

### Ovarian stimulation for IVF

We performed a long gonadotropin releasing hormone agonist (GnRHa) protocol with down regulation using a single dose of 1.3 mg long-acting triptorelin and ovarian stimulation with 225 IU/d of recombinant FSHα (rFSH) and 75 IU/d hMG. When four follicles reached to 20 mm, 19 mm,16 mm and 15 mm in diameter, 10,000 IU of human chorionic gonadotropin (HCG) was administered for triggering the maturation of oocytes on Day 21 of stimulation. On the triggering day serum levels of E_2_ and P were 33 pg/ml and 2.3 ng/ml respectively with a thin endometrium (4 mm), which showed less disorder comparing with them in the previous cycle of ovulation induction without oral dexamethasone and GnRH agonist down regulation. Then 4 oocytes were retrieved 36 h after HCG triggering and 4 cleavage embryos were available and cryopreserved. The details were shown in Table 2.

**Table 2:** Ovarian stimulation for IVF and the changes of hormones

Cycle day	−21	0	6	11	17	23	26
dexamethasone	0.375 mg/d						
long-acting triptorelin (mg)	1.3	/	/	/	/	/	/
rFSH (IU/d)	/	225	150	75	75	/	/
HMG (IU/d)	/	/	75	150	225	300	/
HCG (IU)	/	/	/	/	/	/	10000
Follicles (mm) and (number)	5 (4)	4 (4),3.5 (3)	6.5 (2),4 (4)	9 (2),7 (2), 5 (3)	11 (2),9 (2), 5 (3)	17 (1),15 (1), 10 (2)	22 (1),17 (2), 12 (1)
Endometrial thickness (mm)	5.0	2.3	2.8	4	4.1	5.0	5.0
FSH (IU/L)	14.5	2.8	14.8	13.1	14.7	15.7	16.4
LH (IU/L)	5.6	3.0	1.6	1.1	0.8	0.6	0.6
E_2_ (pg/ml)	20	20	< 20	< 20	< 20	30	33
P (ng/ml)	0.3	0.2	0.1	0.2	0.3	1.6	2.3

*Abbreviation*: r*FSH* Recombinant FSHα, *HMG* Human menopausal gonadotropin, *HCG* Human chorionic gonadotrophin, *FSH* Follicle-stimulating hormone, *LH* Luteinizing hormone, *E*_*2*_ Estradiol, *P* Progesterone

### Pregnancy after frozen-thawed embryo transfer with artificial endometrial preparation

The patient’s menses came 17 days after oocyte retrieval. On cycle 3 serum P level was 0.6 ng/ml and artificial endometrial preparation was started with oral estradiol valerate (4 mg/d). When endometrial thickness reached 10.4 mm, progesterone in oil (60 mg/d) was administered by intramuscular injection, and 3 days later, two frozen-thawed embryos were transferred. After embryo transfer, oral dexamethasone wasn’t given any longer considering the patient had never presented with adrenal insufficiency before. A twin pregnancy was attained and estradiol and progesterone was maintained during the first trimester of pregnancy. The pregnancy proceeded uneventfully, with the regular monitor in the department of Endocrinology and Obstetrics. A healthy boy and a healthy girl were delivered by caesarean section after 37 weeks and 2 days of gestation, weighing 2.5 kg and 2.3 kg respectively. No perinatal problems were observed, and the puerperium was uneventful. During the pregnancy and post-partum period, she had not presented with adrenal insufficiency and no need for glucocorticoids replacement. She remained amenorrhea 1 year after delivery and has been accepting HRT until now.

### Literature search

We searched the PubMed database using search terms for Medical Subject Headings and/or text words relating to P450 oxidoreductase deficiency and pregnancy. The retrieved papers were hand-searched for additional relevant articles using our inclusion criteria (papers reporting *POR* gene mutations in 46, XX females). Only two papers and three female cases with homozygous or compound heterozygous mutations in *POR* gene were reported to be successful pregnant [23, 26]. Including our case, all four successful pregnant cases were obtained by IVF and no spontaneous pregnancy has been reported (Table 3).

**Table 3:** The currently reported pregnant cases in 46, XX females with PORD

Numbers of cases	Ethnicity	age	POR mutation	Amino acid changes	Clinical findings	Hormone status	Fertility therapy and outcome	Reference
1	Chinese (case 1)	28	Homozygous, c.976 T > G	p.Y326D	Primary infertility, irregular menses, frequent ovarian cyst, history of vaginal atresia, absence of clinical hyperandrogenism, no skeletal malformation	ACTH, cortisol and basal P were in normal range but basal 17-OHP were elevated and no obvious sign of adrenal insufficiency . While E_2_ levels were low and P levels were elevated during ovarian stimulation and before ovulation triggering.	IVF and frozen embryo transfer with artificial endometrial preparation after suppressing P by GnRHa and dexamethasone. Singleton live birth.	[26]
2	European (case 2,3)	30,36	Compound heterozygous, c.1249-1G *>* C/ c.1324C *>* T c.1825C *>* T/ c.1859G *>* C	p.(?)/ p.Pro442Ser p.Gln609∗/p.Trp620Ser	Primary infertility, irregular menses, ovarian cyst or history of ovarian cyst, normal genitalia, absence of clinical hyperandrogenism, no skeletal malformation (the same findings in both cases)	ACTH, cortisol and basal P levels were in normal range, the response of cortisol to ACTH stimulation was insufficient. While E_2_ levels were modestly increased and P levels were increased in the range of luteal phase during ovarian stimulation and before ovulation triggering.	IVF and frozen embryo transfer after suppressing P by hydrocortisone (case 2) and dexamethasone (case 3) while no mention of the endometrial preparation protocol. Twins live birth (case 1) and singleton live birth (case 2)	[23]
1	Chinese	29	Compound heterozygous, c.1370G > A/ c.1196_1204del	p.Arg457His, rs28931608/ p.Pro399_Glu401del	Primary infertility, primary amenorrhea, frequent ovarian cyst, hypoplastic uterus, absence of clinical hyperandrogenism, mild skeletal malformation	ACTH, cortisol and basal P were in normal range but basal 17-OHP were elevated and no obvious sign of adrenal insufficiency. . While E_2_ levels were low and P levels were elevated during ovarian stimulation and before ovulation triggering.	IVF and frozen embryo transfer with artificial endometrial preparation after suppressing P by dexamethasone. Twins live birth.	This paper

*Abbreviation*: *E*_*2*_ Estradiol, *P* Progesterone, *17-OHP* 17α-hydroxyprogesterone, *ACTH* Adrenocorticotropic hormone, *IVF* In vitro fertilization, *GnRHa* Gonadotrophin releasing hormone agonist

## Discussion

A recently published review showed that PORD is a complex disorder with many possible mutations affecting a large number of enzymes and the most common mutations were R457H(25%) and A287P(24%) in 180 individual POR mutations from 90 patients [7]. Several phenotypic features were very common in PORD women but occurred across a range of mutations, including of high serum concentrations of P (100%), pregnenolone (100%), 17OHP (96%), corticosterone (83%) and deoxycorticosterone (70%), DSD (78%), ovarian cysts (39%), skeletal malformations (84%), and adrenal insufficiency (78%) with most of mild cases [7]. For late-onset PORD primary amenorrhea/oligomenorrhea or infertility could be the main clinical manifestation [2, 23, 25], but little is known about the optimal way to investigate and treat patients with adult-onset PORD. Our case was a late-onset PORD woman presented with features of high basal serum P and 17OHP, primary amenorrhea, ovarian cyst, minor skeletal malformation and no obvious sign of adrenal insufficiency. A compound heterozygotes for c.1370G > A (R457H) and c.1196_1204del (P339_E401del) were found which had been confirmed to reduce activities of P450c17, P450c21, and P450aro [5, 11, 12, 15, 22]. As so far the clinical course and of PORD in adulthood and the long-term consequence for fertility remain unknown. We searched the literature from PubMed database and found that up to now no spontaneous pregnancy and only two reports of three successful pregnancies after IVF have been reported in PORD female patients [23, 26]. Comparing with our case the three reported cases presented with milder phenotypes (Table 3): case 1 showed oligomenorrhea, ovarian cyst and normal serum 17OHP (basal serum P was not mentioned) [26]; case 2 and 3 showed oligomenorrhea, ovarian cyst, high serum P and 17OHP [23]; the three cases and our case showed different mutations of *POR* gene. Therefore, at least until now all these pregnant cases should be the non-classic form of PORD without obvious sign of adrenal insufficiency.

All three reported pregnant cases presented with primary infertility and had accepted IVF treatment. Letrozole combined with hMG protocol in case 1, GnRH agonist and GnRH antagonist protocols in case 2 and 3 were used for IVF (Table 3) [23, 26]. During FSH/hMG stimulation and on the day of ovulation triggering, the three cases showed normal follicular growth but only modestly increasing estradiol (37.09, 90 and 30 pg/ml respectively, compared with 10-fold higher levels in common IVF cycles) and unusual increasing serum P levels to the range of luteal phase (Table 3) [23, 26]. While 2, 9 and 15 oocytes were retrieved respectively and fresh embryo transfer were all cancelled due to thin endometrium and serum P elevation (Table 3) [23, 26]. These similar hormone changes during ovarian stimulation also occurred in our case. During the ovulation induction with hMG she presented with normal follicular growth but higher serum P and thin endometrium with undetectable serum E_2_, which means that ovarian stimulation and follicular development increase the P overproduction from ovary. These non-classic PORD female patients who lack the genital and obvious skeletal malformations are usually undiagnosed in their early age, just like these reported cases and our case. These cases remind reproductive gynecologists and endocrinologists to consider the possibility of non-classic PORD when patients present with amenorrhea, oligomenorrhea, unexplained infertility, the presence of ovarian cysts, high basal P or 17OHP and a specific pattern of response to ovarian stimulation. Our case provided additional information of effective infertility treatment in PORD women with different ethnicity, clinical phenotype and *POR* gene mutation. The impairment of reproductive capacity in non-classic PORD women may be mainly explained by the effects of estradiol deficiency and progesterone excess from both adrenal and gonad, accentuated by ovarian stimulation, on endometrial development.

IVF can be used to segment ovarian stimulation and embryo transfer to avoid the negative effect of high P and low E_2_ on endometrial receptivity by freezing all available embryos. Freeze-all policy have been successfully used in women undergoing IVF under various conditions such as the premature elevation of serum P after conventional ovarian simulation [16, 19], luteal phase stimulation [19, 27] and progestin-primed ovarian stimulation protocol and so on [16, 28]. Therefore, it suggests that high P level during the period of follicular growth may not impair the developmental capacity of the oocyte and the effect of high P on endometrium can be overcome with cryopreservation and frozen-thawed embryo transfer (FET). As for low estradiol, previous reports showed estrogen may not play a key role in folliculogenesis and follicular development in vivo and in vitro [14, 20], but gonadotrophins play a vital role in the growth and maturation of follicles [10]. In the successful pregnancies in CAH women caused by 17-hydroxylase deficiency and steroidogenic acute regulatory protein mutations, the patients presented with primary amenorrhea and absent or incomplete sexual maturation [1, 4]. The authors both reported that during their IVF treatment, endogenous estrogen level was very low but follicles grew normally after ovarian stimulation and normal embryos and pregnancies were obtained [1, 4], just as the successful pregnant PORD cases and our case reported (Table 3). So we suggest that the disorders of gonadal steroidogenesis caused by rare forms of CAH may have little effect on the follicular growth and the developmental capacity of the oocytes. When the embryos of the PORD cases were obtained after IVF, embryo transfer was performed with artificial endometrial preparation protocol when optimal P suppression to the normal range of follicular phase by glucocorticoids (dexamethasone 0.75 mg, hydrocortisone 25–30 mg, dexamethasone 0.5 mg per day in reported case 1, 2 and 3) (Table 3) [23, 26]. Dexamethasone 0.375 mg per day and artificial endometrial preparation protocol were also used in our case. Although only few cases have been reported to be successful pregnancies, it may be an effective way to help them have their own children, through IVF and FET after using exogenous estrogen for endometrial preparation and corticoids to suppress the overproduction of progesterone. Of course, a multidisciplinary team including reproductive endocrinologist, internal endocrinologist, obstetrician and geneticist is needed for these women to get through the pregnancy and delivery [6].

## Conclusions

In conclusion, It is a third report of successful pregnancy in a PORD patient who had primary amenorrhea and different *POR* mutation with the published three cases who had been reported to obtain successful pregnancies after IVF. For this rare form of CAH, it seemed that disorders of steroidogenesis caused by PORD didn’t impair the developmental potential of oocytes. The successful pregnancy could be obtained through IVF and FET after adequate hormonal control and endometrial preparation. Our report will hopefully improve the timely diagnosis and effective treatment of infertility in PORD women.

## Data Availability

The data used and/or analyzed during the current study are available from the corresponding author on reasonable request.

## Abbreviations

CAH: Congenital adrenal hyperplasia
PORD: P450 oxidoreductase deficiency
POR: P450 oxidoreductase
E_2_: Estradiol
P: Progesterone
IVF: In vitro fertilization
NADPH: Nicotinamide adenine dinucleotide phosphate
DSD: Sexual development
HRT: Hormone replacement therapy
HMG: Human menopausal gonadotropin
17-OHP: 17-hydroxyprogesterone
DHEA-S: Dehydroepiandrosterone sulfate
NGS: Targeted exome next-generation sequencing
ACMG: The American College of Medical Genetics and Genomics
HCG: Human chorionic gonadotropin
GnRHa: Gonadotropin releasing hormone agonist
FET: Frozen-thawed embryo transfer
